# Endoscopic retrograde cholangiopancreatography in the elderly: a review of most recent personal experience

**DOI:** 10.1186/1471-2482-13-S1-A13

**Published:** 2013-09-16

**Authors:** Giovanni D De Palma, Bruno Amato, Saverio Siciliano, Francesco Maione, Dario Esposito, Nicola Gennarelli, Marcello Persico, Stefania Masone, Giuseppe Iannone, Pietro Forestieri

**Affiliations:** 1 Department of Gastroenterology, Endocrinology and Surgery. Center of Excellence for Technical Innovation in Surgery, University of Naples Federico II, School of Medicine, Italy

## Introduction

It is well documented that the incidence of biliary tract pathologies increases with age, and as life expectancy is rising, it is expected that the prevalence of advanced age patients with bile duct pathologies will correspondingly increase leading to an increase in the demand for endoscopic retrograde cholangiopancreatography (ERCP) interventions.

Our aims are to assess the outcomes, safety and complications associated with ERCP performed in an elderly population.

## Methods

Patients aged 80 years or over referred for ERCP from January 2008 to December 2012, were identified and retrospectively reviewed.

## Results

Eighty-three patients (58 females, mean age 84.6 ± 3.9 years old) underwent 94 procedures (1-3 procedures/patient). The main indications were cholangitis (53.7%), choledocholithiasis (19.4 %) and blocked stents (14.6 percent). Malignancies represented 12.3% of indications. The overall success rate was 92.5%. Minor events occurred in 19 patients (22.9%) percent (tachycardia 5, desaturation 11, transient hypotension 3). Major events occurred in 4 patients (4.8%): delayed post sphincterotomy bleeding (five days post-procedure) in 1 patient, cholangitis in 2, and one mild pancreatitis. Four deaths occurred within one month of ERCP, due to advanced malignancies.

## Conclusion

Our study showed that ERCP is safe in the elderly population. Minor complications are usually transient and related to sedation, and mortality is usually related to severity of illness and underlying malignancies.
